# Discovery of the Ligament of Modiolus: Anatomical Insights and Clinical Relevance

**DOI:** 10.1007/s00266-025-04696-0

**Published:** 2025-02-03

**Authors:** Kyu-Ho Yi, Jovian Wan, Song-Eun Yoon, Hugues Cartier, Sebastien Garson, Benrita Jitaree, Soo-Bin Kim

**Affiliations:** 1 Division in Anatomy and Developmental Biology, Department of Oral Biology, Human Identification Research Institute, BK21 FOUR Project, Yonsei University College of Dentistry, 50-1 Yonsei-ro, Seodaemun-gu, Seoul, 03722 Korea; 2 Maylin Clinic (Apgujeong), Seoul, Korea; 3 Medical Research Inc., Wonju, Korea; 4 Brandnew Aesthetic Surgery Clinic, Seoul, Korea; 5 Centre Médical Saint Jean, Arras, France; 6 Cabinet Médical, Senlis, France; 7 Faculty of Medicine Ramathibodi Hospital, Chakri Naruebodindra Medical Institute, Mahidol University, 111, Bang Pla, Bang Phli, Samut Prakan, 10540 Thailand; 8 Department of Oral Anatomy, Institute of Biomaterial Implant, College of Dentistry, Wonkwang University, Iksan, 54538 Korea

**Keywords:** Ligament of modiolus, Aging anatomy, Modiolus anatomy, False ligament, Perioral dynamics, Midface reconstruction, Cadaveric study

## Abstract

The modiolus is a critical anatomical structure in facial expressions and oral competence, serving as a hub where multiple muscles converge. Our recent cadaveric study has identified a previously unreported structure, the ligament of modiolus, aligning with the line of ligament between the modiolus and its adjacent muscular and fascial attachments. This novel finding offers new perspectives on the anatomical organisation of the perioral region and its functional significance. Importantly, it has been identified as a false ligament rather than a true ligament, originating from the modiolus and extending to the dermal layer. The discovery has potential implications for aesthetic and reconstructive procedures involving the midface and oral commissures. Here, we present our findings and discuss their relevance to facial anatomy, surgical approaches, and clinical outcomes.

*Level of Evidence V* This journal requires that authors assign a level of evidence to each article. For a full description of these Evidence-Based Medicine ratings, please refer to the Table of Contents or the online Instructions to Authors  www.springer.com/00266.

## Introduction

The modiolus is a fibromuscular structure at the angle of the mouth, where multiple facial muscles, including the orbicularis oris, buccinator, and zygomaticus major, converge. It plays a pivotal role in dynamic lip movement, oral competence, and facial expressions [1–3]. While extensively studied, the anatomical components of the modiolus have remained incompletely characterised.

Our cadaveric dissection has uncovered a ligamentous component within the modiolus, termed the conjoined fibromuscular tissue mass of modiolus (ligament of modiolus), which aligns with the known line of ligament connecting neighbouring anatomical structures. However, this ligament has been determined to be a false ligament, originating from the modiolus and terminating in the dermal layer, rather than a true ligament connecting to bony or deep fascial structures. This discovery enhances our understanding of the modiolus as both a functional and structural entity. The finding has profound implications for facial surgery, where preserving or reconstructing the modiolus is critical for maintaining perioral dynamics.

## Methods

### Cadaveric Study Design

**Fig. 1:**
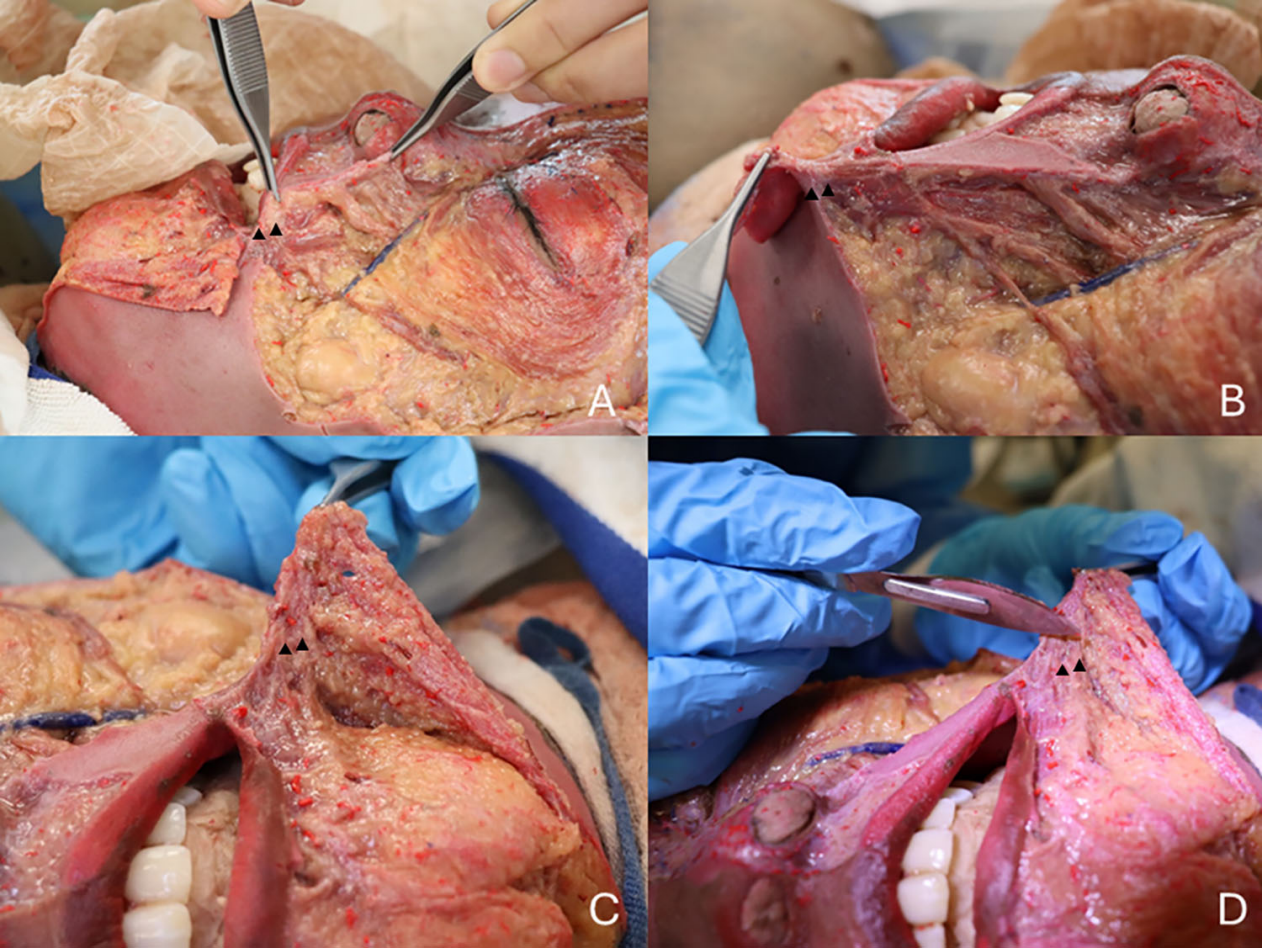
Images showing different angles of the conjoined fibromuscular tissue mass of modiolus identified in cadaveric dissection. Panels **A** and **B** display lateral views, highlighting the alignment of the ligament with the line of ligament. Panels **C** and **D** provide anterior views, illustrating the ligament's span from the modiolus to the dermal layer. The arrows indicate the conjoined fibromuscular tissue mass of modiolus

Dissections were conducted on six cadavers (four males and two females) of varying ethnicities. Bilateral dissections of the midface were performed to expose the modiolus and its associated structures. The ligament was identified through meticulous dissection and visualised under magnification. The findings were documented photographically and are presented in Fig. 1, showing the ligament from multiple angles to illustrate its structural and anatomical context.

In this study, meticulous dissections were performed on six cadavers to investigate the anatomical structure of the modiolus and its associated components. The cadavers, ranging in age from 21 to 68 years (21, 34, 45, 52, 61, and 68 years old), were dissected laterally to medially, with the skin removed in even layers to preserve the integrity of the underlying structures. This approach allowed for detailed visualisation and precise identification of the ligamentous features within the modiolus. Demographic information, including age and sex, was documented to provide context for the findings and enhance the reproducibility of the research. The dissection process was systematically conducted to maintain consistency across all specimens, ensuring reliable comparisons and observations.

## Result

### Anatomical Description

The conjoined fibromuscular tissue mass of modiolus was consistently identified in all six cadavers dissected, revealing its pivotal role in perioral anatomy. It was observed to span from the modiolus to the dermal layer, reinforcing its classification as a false ligament. This structure forms a vital component of the superficial framework of the midface, directly contributing to the stability of the modiolus as a hub for muscle attachments (Fig. 1).

The ligament aligns with the "line of ligament," an anatomical continuity that links the orbicularis oris, buccinator, and zygomaticus major to superficial supportive structures. Its composition was distinct from surrounding tissues, consisting of dense collagenous fibres interspersed with elastic components, as highlighted in Fig. 1, which demonstrates the ligament’s alignment and integration within the perioral region. This unique arrangement imparts both strength and flexibility, allowing the ligament to anchor the modiolus effectively while accommodating dynamic facial movements.

## Functional Orientation

By anchoring the modiolus to the dermal layer, the ligament stabilises the perioral region, ensuring effective transmission of forces generated by muscle contractions. This stabilisation facilitates precise movements of the lips, such as puckering, smiling, and speech articulation, while maintaining the integrity of the oral commissures.

## Clinical Relevance

Preservation of the ligament during reconstructive and aesthetic procedures involving the midface or oral commissures appears critical for optimising both function and aesthetics. The ligament's role in maintaining oral sphincteric function and preventing drooping of the oral commissures highlights its importance. In cases of modiolus reconstruction, ensuring the continuity of the ligament may enhance long-term surgical outcomes by preserving natural lip dynamics and oral competence.

## Discussion

The discovery of the conjoined fibromuscular tissue mass of modiolus redefines the modiolus as not only a fibromuscular hub but also a false ligamentous anchor within the facial architecture. This ligament’s alignment with the line of ligament underscores the structural continuity in the midface, providing a deeper understanding of perioral dynamics. Its integration with both muscular and fascial components suggests a critical role in linking superficial and deep structures to facilitate coordinated movements of the perioral region.

In the context of aesthetic surgery, this finding has profound implications. Facial rejuvenation procedures targeting the nasolabial folds and oral commissures should account for the ligament’s role in maintaining structural integrity. Techniques such as dermal fillers or fat grafting must avoid disrupting this ligament to preserve natural expressions and prevent complications such as asymmetry or drooping. Similarly, in reconstructive surgery, the ligament plays a crucial role. Reconstruction of large lip or midface defects must prioritise preserving or recreating the ligament to ensure both functional and aesthetic outcomes. Surgical techniques, including Bernard–Webster or Karapandzic flaps, could benefit from modifications that incorporate the ligament’s anatomy. Understanding the ligament’s role in stabilising the modiolus and transmitting forces can refine surgical approaches, optimising recovery and minimising complications.

The study’s cadaveric nature presents some limitations. Although the ligament’s structural presence was consistently observed, its functional significance in live subjects requires further investigation. Advanced imaging techniques could provide in vivo visualisation, aiding in preoperative planning and anatomical education. Moreover, exploring the ligament’s role in pathological conditions, such as facial paralysis or trauma, could expand its clinical relevance. Future research should also investigate the biomechanics of this ligament in dynamic facial movements, offering insights into its role in both health and disease.

## Conclusion

The conjoined fibromuscular tissue mass of modiolus has been identified as a false ligament, originating from the modiolus and extending to the dermal layer. This newly described structure contributes significantly to the stability and functionality of the perioral region, providing key insights into the anatomical organisation of the midface. Preservation of this ligament during surgical procedures offers the potential to enhance both functional and aesthetic outcomes. Further studies are warranted to explore its role in dynamic facial movements and pathological conditions, paving the way for innovative surgical techniques.
